# Spider venom peptides Ht1a and Gg1a are toxic to honeybee parasite *Varroa destructor* by topical application

**DOI:** 10.1038/s44386-026-00050-9

**Published:** 2026-06-03

**Authors:** Volker Herzig, Shaodong Guo, David A. Eagles, Sandy S. Pineda, Alexandra Robinson, Asa Andersson, Jennifer Deuis, Zoltan Dekan, Paul F. Alewood, Eivind A. B. Undheim, Maxime Lammens, Frank Bosmans, Irina Vetter, Glenn F. King, Vincent Dietemann

**Affiliations:** 1 Centre for Bioinnovation, University of the Sunshine Coast, Sippy Downs, QLD Australia; 2 School of Science, Technology and Engineering, University of the Sunshine Coast, Sippy Downs, QLD Australia; 3 Institute for Molecular Bioscience, The University of Queensland, Brisbane, QLD Australia; 4 Centre for Ecological and Evolutionary Synthesis, Department of Biosciences, University of Oslo, Oslo, Norway; 5 Department of Basic and Applied Medical Sciences, Ghent University, Ghent, Belgium; 6 Experimental Pharmacology Group, Faculty of Medicine and Pharmaceutical Sciences, Vrije Universiteit Brussel, Brussels, Belgium; 7 School of Pharmacy and Pharmaceutical Sciences, The University of Queensland, Brisbane, QLD Australia; 8 Australian Research Council Centre of Excellence for Innovations in Peptide and Protein Science, The University of Queensland, Brisbane, QLD Australia; 9 Swiss Bee Research Centre, Agroscope, Bern, Switzerland; 10 Department Ecology and Evolution, University of Lausanne, Lausanne, Switzerland; 11 Present Address: Brain and Mind Centre, and School of Medical Sciences, Faculty of Medicine and Health, University of Sydney, Camperdown, NSW Australia

**Keywords:** Biochemistry, Chemical biology, Ecology, Ecology, Zoology

## Abstract

Global food supply strongly depends on honeybee pollination services, which are threatened by insecticides and pests such as parasitic *Varroa destructor* mites. Chemical varroacides/acaricides are hampered by resistance development, necessitating the development of sustainable and environmentally friendly alternatives, with arthropod venom peptides being considered promising sources of acaricidal toxins. With only a few acaricidal venom peptides being reported, we performed a systematic topical screening of 50 arthropod venoms against *V. destructor*, with 78% of the venoms causing 100% mortality after 24 h. Deconvolution of the venoms from the Tasmanian cave spider *Hickmania troglodytes* and the Giant Japanese funnel-web spider *Gigathele gigas* led to identification of the varroacidal peptides Ht1a and Gg1a. Topical application of Ht1a and Gg1a reduced varroa mite but not honeybee survival, despite Ht1a inhibiting voltage-gated sodium channels from varroa and honeybee with equal potency. Ht1a and Gg1a were inactive against human skeletal muscle (hNa_V_1.4), cardiac (Na_V_1.5), neuronal Na_V_ channel isoforms, and human voltage-gated calcium channel Ca_V_2.2. At human α3β2/4 nicotinic acetylcholine receptors, Gg1a was inactive while 10 µM of Ht1a partially blocked nicotine-mediated Ca^2+^ influx. Our data reveal Ht1a and Gg1a as promising candidates for the development of novel varroa mite treatments of honeybee hives.

## Introduction

A third of the world’s food supply results directly or indirectly from insect pollination, with an annual value of EUR 153 billion^1,2^. Honeybees are one of the world’s major managed insect pollinators. They increase the yield of a wide variety of highly nutritious crops and are therefore crucial for global food security and human health^3–5^. Unfortunately, honeybees are facing multiple threats from a variety of factors, including chemical insecticides, food shortage due to wildflower loss, viruses, and parasites^6^. Parasitic *Varroa destructor* mites are currently considered the major threat for apiculture, with up to 85% of *Apis mellifera* colony losses directly linked to infestation by this parasite^7^, which is a known vector of bee viruses that are also linked to colony loss^8^. With the recent invasion of *V. destructor* into Australia in 2022 and the subsequent unsuccessful eradication attempts, it has now become a truly global problem for *A. mellifera* apiculture^9^. Synthetic acaricides (i.e., pesticides that target ticks and mites) currently used to control *V. destructor* include the formamidine amitraz (octopamine receptor agonist), the organophosphate coumaphos (acetylcholinesterase inhibitor), and the pyrethroids flumethrin and fluvalinate (voltage-gated sodium channel agonists)^10^. Unfortunately, some *V. destructor* populations have already acquired resistance to these acaricides^11–13^. Moreover, acaricides can pollute honey and wax, leading to further economic losses for beekeepers. New bee-friendly treatment options for *V. destructor* infestations are therefore urgently needed^14–16^.

A possible alternative solution for control of *V. destructor* is venom-derived acaricidal peptides. Venomous arachnids such as spiders and scorpions prey on arthropods, including other arachnids^17–19^. It is therefore unsurprising that the wide-spectrum insecticidal spider-venom peptide ω-hexatoxin-Hv1a (hereafter Hv1a) is also acaricidal^20^. However, somewhat unexpectedly, Hv1a is inactive against honeybees and mammals^21,22^. It therefore seems plausible that within the venoms of >53,000 extant spider^23^ and >2,800 extant scorpion species^24^, each comprising hundreds to thousands of peptide toxins^25^, there might be some peptides that are selectively toxic to *V. destructor* but safe for beneficial species and humans. The fact that varroa mites spend most of their time attached to honeybees in infected colonies renders traps containing acaricidal components less effective, with topical application strategies likely to be more effective. Thus, in the present study, we systematically screened 50 arthropod venoms (mainly from spiders and scorpions) to identify peptides with topical varroacidal activity. We identified two spider-venom peptides with promising varroacidal activity and further characterised their activities against varroa mites, honeybees, and key off target human ion channels and receptors.

## Results and discussion

### Varroacidal activity is widespread in arthropod venoms

While many arthropod venom peptides with insecticidal activity have been reported^26,27^, peptides with reported activity against arachnids (e.g., spiders, scorpions or mites) are still rare^18,20^. This might be due to researchers being biased towards testing insecticidal activities, as pest insects are generally easier than arachnids to culture. To discover novel acaricidal venom peptides, we set up a semi-automated assay to determine the activity of 50 taxonomically diverse arthropod venoms against *V. destructor* mites. At a dose of 10 μg venom topically applied to each *V. destructor* mite, 100% *V. destructor* mortality was caused by 13 venoms (26%) after 2 h, by 17 venoms (34%) after 6 h, and by 39 venoms (78%) after 24 h (Table 1). This widespread topical activity is rather surprising given that arthropods deliver their venom directly into the body of their victims through bites or stings. The surfactant Silwet was not used for the initial topical screening of the venoms against varroa mites. Therefore, the abundantly observed varroacidal activity must have been induced by the arthropod venoms. The mechanism by which varroacidal peptides penetrate the varroa cuticle still remains to be determined, which could, for example, be via softer parts of the cuticle or through the spiracles. Entry via the spiracles has already been suggested for some insecticidal spider-venom peptides^28^.

**Table 1:** Varroacidal activity of arthropod venoms.

% dead mites at						
**2** **h**	**6** **h**	**24** **h**	**Genus**	**Species**	**Family**	**Organism**
**100**	**100**	**100**	*Atrax*	*robustus*	Atracidae	spider
**100**	**100**	**100**	***Hickmania***	***troglodytes***	**Gradungulidae**	**spider**
**100**	**100**	**100**	*Linothele*	*fallax*	Dipluridae	spider
**100**	**100**	**100**	***Gigathele***	***gigas***	**Macrothelidae**	**spider**
**100**	**100**	**100**	*Nephila*	*pilipes*	Nephilidae	spider
**100**	**100**	**100**	*Avicularia*	*cf aurantiaca*	Theraphosidae	spider
**100**	**100**	**100**	*Avicularia*	*cf juruensis*	Theraphosidae	spider
**100**	**100**	**100**	*Avicularia*	*purpurea*	Theraphosidae	spider
**100**	**100**	**100**	*Avicularia*	*spec.(“huriana”)*	Theraphosidae	spider
**100**	**100**	**100**	*Avicularia*	*spec.(“AP”)*	Theraphosidae	spider
**100**	**100**	**100**	*Avicularia*	*spec.(“Tambopata”)*	Theraphosidae	spider
**100**	**100**	**100**	*Avicularia*	*variegata*	Theraphosidae	spider
**100**	**100**	**100**	*Caribena*	*versicolor*	Theraphosidae	spider
78	**100**	**100**	*Avicularia*	*spec.(“pupurea wrong”)*	Theraphosidae	spider
78	**100**	**100**	*Avicularia*	*spec.(“Al”)*	Theraphosidae	spider
75	**100**	**100**	*Leiurus*	*quinquestriatus deserti*	Buthidae	scorpion
20	**100**	**100**	*Leiurus*	*quinquestriatus*	Buthidae	scorpion
80	80	**100**	*Linothele*	*megatheloides*	Dipluridae	spider
80	80	**100**	*Nephila*	*plumipes*	Nephilidae	spider
78	78	**100**	*Eriophora*	*transmarina*	Araneidae	spider
78	78	**100**	*Megadolomedes*	*australianus*	Pisauridae	spider
56	78	**100**	*Isopeda*	*villosa*	Sparassidae	spider
40	78	**100**	*Cupiennius*	*salei*	Ctenidae	spider
20	78	**100**	*Androctonus*	*australis*	Buthidae	scorpion
11	78	**100**	*Polybetes*	*pythagoricus*	Sparassidae	spider
56	75	**100**	*Avicularia*	*spec.(“Amazone Purple”)*	Theraphosidae	spider
60	60	**100**	*Diplura*	*spec*.	Dipluridae	spider
0	60	**100**	*Phoneutria*	*nigriventer*	Ctenidae	spider
20	56	**100**	*Parabuthus*	*villosus*	Buthidae	scorpion
0	40	**100**	*Phoneutria*	*reidyi*	Ctenidae	spider
11	33	**100**	*Neosparassus*	*diana*	Sparassidae	spider
0	33	**100**	*Havinthus*	*rufovarius*	Reduviidae	assassin bug
0	33	**100**	*Heterometrus*	*cf cyaneus*	Scorpionidae	scorpion
20	20	**100**	*Nebo*	*hierichonticus*	Diplocentridae	scorpion
20	20	**100**	*Allocosa*	*obscuroides*	Lycosidae	spider
17	17	**100**	*Ancylometes*	*spec*.	Ctenidae	spider
11	11	**100**	*Heteropoda*	*jugulans*	Sparassidae	spider
0	0	**100**	*Viridasius*	*fasciatus*	Viridasiidae	spider
0	0	**100**	*Viridasius*	*spec.(sylvestriformis)*	Viridasiidae	spider
75	75	71	*Avicularia*	*spec.(‘E”)*	Theraphosidae	spider
71	71	60	*Ybyrapora*	*diversipes*	Theraphosidae	spider
0	0	60	*Urodacus*	*manicatus*	Urodacidae	scorpion
0	11	50	*Sparassidae*	*spec.(Flores)*	Sparassidae	spider
20	56	43	*Ancylometes*	*spec*.	Ctenidae	spider
33	50	43	*Ephebopus*	*cyanognathus*	Theraphosidae	spider
20	33	14	*Phoneutria*	*fera*	Ctenidae	spider
0	11	14	*Ancylometes*	*rufus*	Ctenidae	spider
0	20	0	*Hadronyche*	*infensa*	Atracidae	spider
11	11	0	*Heterometrus*	*spinifer*	Scorpionidae	scorpion
0	0	0	*Pristhesancus*	*plagipennis*	Reduviidae	assassin bug

A panel of 50 arthropod venoms were topically applied to *V. destructor* mites at 10 μg/venom/mite (*n* = 5 mites/venom). Percentage mortality was calculated at 2, 6 and 24 h, and adjusted for control mortality. 100% mortality is highlighted in bold. Data are sorted by decreasing mortality at 24 h. Venoms of two spiders from the less well studied families Gradungilidae and Macrothelidae (indicated in bold) were selected for further purification of the active varroacidal venom peptides.

### Isolation, purification and sequencing of varroacidal venom peptides from two spider families

Based on available venom quantities and to maximise taxonomic diversity, two venoms from the initial venom screen (Table 1) that caused 100% varroa mortality 2–24 h after venom application were selected for further isolation of varroacidal peptides. These venoms were from the Tasmanian cave spider *Hickmania troglodytes* (family Gradungulidae) and the Giant Japanese funnel-web spider *Gigathele gigas* (family Macrothelidae). *G. gigas* is a ‘primitive’ mygalomorph spider, whereas *H. troglodytes* are long-lived cribellate araneomorph spiders found only in Tasmania.

**Fig. 1:**
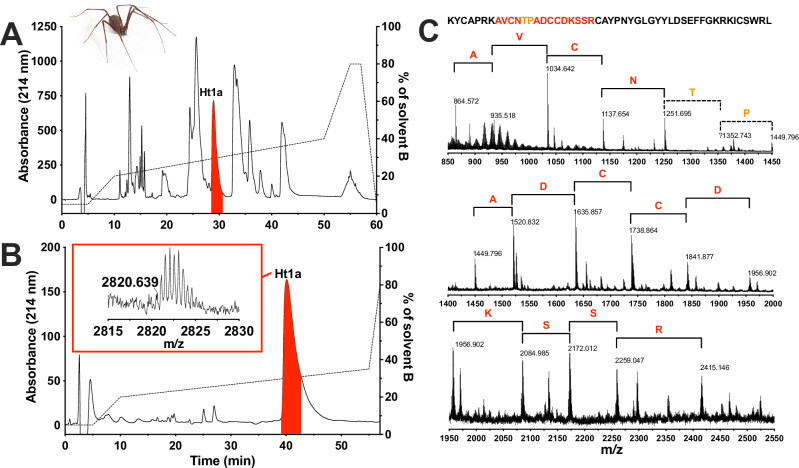
Isolation and sequencing of Ht1a. **A** Chromatogram resulting from C18 RP HPLC fractionation of pooled venom from female Tasmanian cave spiders (*H. troglodytes*, pictured top left). **B** Chromatogram resulting from purification of the red fraction highlighted in panel A using a narrow-bore C18 RP HPLC column. Inset shows the MALDI-MS spectrum of purified Ht1a with m/z indicated for the double-charged ion, corresponding to an uncharged monoisotopic molecular mass of 5639.278 Da. The red peak contains the varroacidal Ht1a peptide. Dashed lines in panels A and B indicate the gradient of solvent B (90% acetonitrile/0.1% FA). **C** C-ions from tandem MS/MS data used to determine a partial sequence tag for Ht1a, corresponding to the coloured residues in the Ht1a sequence at top. Red indicates confident assignments, while orange indicates tentative assignments (as no signal at *m*/z of 1352.743 was detected). The remaining sequence information (in black) was identified from the *H. troglodytes* venom-gland transcriptome. *H. troglodytes* photo: EABU.

Fractionation of *H. troglodytes* venom using C18 RP HPLC revealed a moderately complex venom (Fig. 1A). Screening of individual venom HPLC fractions in the varroa assay led to the identification of a single peak (highlighted in red in Fig. 1A) with topical varroacidal activity. Further deconvolution of this fraction revealed a dominant peptide with a monoisotopic molecular mass of 5639.28 Da that contained the varroacidal activity (highlighted in red in Fig. 1B). MALDI-ISD-MS of Ht1a revealed a partial 15-residue sequence tag (Fig. 1C, coloured residues), which was used to search the transcriptome assembled from *H. troglodytes* venom glands (ENA accession number PRJEB15860). This approach gave a single match to a complete prepropeptide, with the mature toxin domain highlighted in bold (also see Fig. 1C):

MKHVSTIEEDDVSHYVEEPEIPSWLEKQVKDHVQDFLATLDPTFANDAELQDMVLAQIEDFLSKKPSPVEEADEPTAR**KYCAPRKAVCNTPADCCDKSSRCAYPNYGLGYYLDSEFFGKRKICSWRL**

The toxin was named μ-gradutoxin-Ht1a (μ-GDTX-Ht1a, hereafter Ht1a) according to the rational nomenclature devised for peptide toxins^29^. The predicted monoisotopic molecular mass of the oxidised mature peptide (highlighted in bold) is 5639.628 Da, which closely matches that of native Ht1a purified from venom (5639.278 Da; Fig. 1B).

**Fig. 2:**
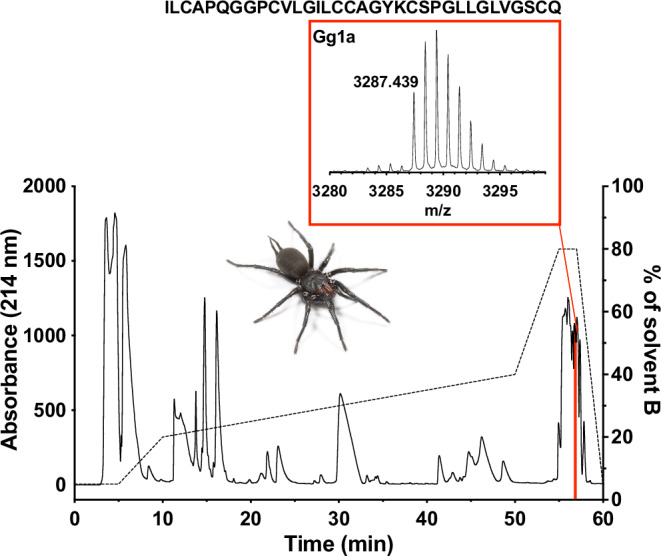
Isolation and sequencing of Gg1a. Chromatogram resulting from C18 RP HPLC fractionation of venom from the funnel-web spider *G. gigas* (photo by Bastian Rast). Dashed line indicates the gradient of solvent B (90% acetonitrile/0.1% FA). The red peak contains the varroacidal Gg1a peptide. Inset shows the MALDI MS spectrum of Gg1a, which revealed an uncharged monoisotopic molecular mass of 3286.439 Da. The primary sequence of Gg1a obtained by Edman sequencing is indicated at the top.

Fractionation of *G. gigas* venom using C18 RP HPLC also revealed a moderately complex venom (Fig. 2). Screening of individual venom HPLC fractions in the varroa assay led to the identification of a single peak with a monoisotopic molecular mass of 3286.439 Da (highlighted in red in Fig. 2) with topical varroacidal activity. We named this peptide U_1_-macrothelitoxin-Gg1a (U_1_-MATX-Gg1a; hereafter Gg1a). N-terminal Edman sequencing of Gg1a returned the following 34-residue peptide sequence: ILCAPQGGPCVLGILCCAGYKCSPGLLGLVGSCQ. The predicted molecular mass for the monoisotopic oxidised mass of the sequence obtained by Edman is 3286.587 Da, which is only 0.148 Da higher than the mass of the native Gg1a. We therefore conclude that the sequence obtained by Edman degradation represents the complete sequence of Gg1a. As for Ht1a, Gg1a has a non-amidated C-terminus.

### In vivo characterisation of sHt1a and sGg1a against *Varroa destructor* and honeybees

**Fig. 3:**
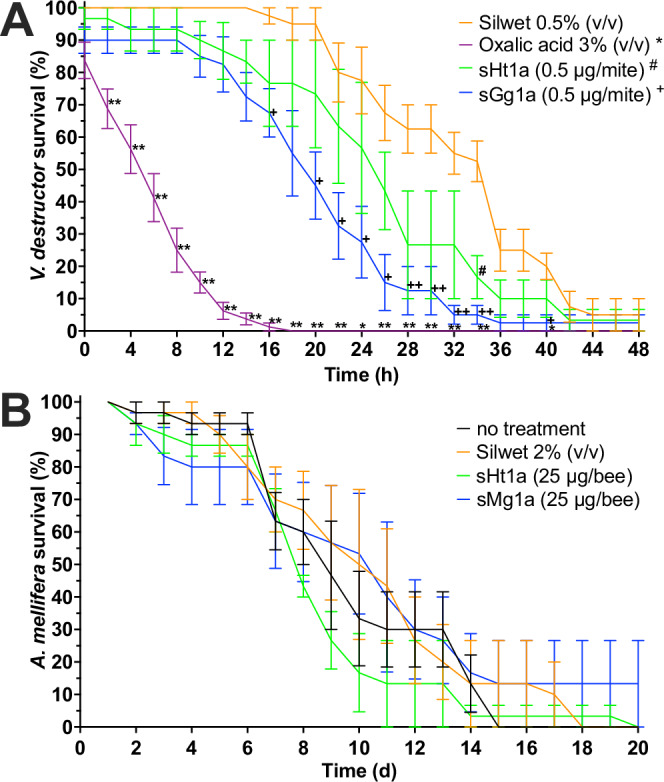
Activity of sHt1a and sGg1a against *Varroa destructor* mites and honeybees, *Apis mellifera.* **A** Topical application of sHt1a, sGg1a and oxalic acid induced time-dependent mortality in *V. destructor* mites. The venom peptides were dissolved in 0.5% Silwet (v/v), which was used as negative control, while oxalic acid was used as positive control. A two-way ANOVA followed by Dunnett’s multiple comparisons test revealed significantly reduced *V. destructor* survival for each treatment (compared to the Silwet group) as indicated by *P < 0.05; **P < 0.01 for oxalic acid, ^#^*P* < 0.05 for sHt1a, and ^+^*P* < 0.05; ^++^*P* < 0.01 for sGg1a. **B** Topical application of a 52-fold higher dose of sGg1a and sHt1a than the dose that caused lethality to varroa mites did not alter honeybee survival relative to the 2% Silwet (v/v) control. All data points are mean ± SEM.

As the available venom quantities limited the amounts of native Ht1a and Gg1a to be isolated from the venoms, milligram quantities of synthetic Ht1a (sHt1a) and Gg1a (sGg1a) were produced by SPPS to facilitate further characterisation. The peptides were applied topically at various concentrations to *V. destructor* mites (*n* = 5 mites per group, and 3–8 repeats per treatment group). Both peptides at 0.48 μg/mite significantly reduced the survival of *V. destructor* compared to the control group receiving 0.5% of the surfactant Silwet (v/v), although with a slower onset than the positive control group that received a 3% oxalic acid solution, which is a commercially used varroacide (Fig. 3A). While oxalic acid caused significant mortality after 2 h, the earliest onset of mortality for the venom peptides was 16 h for sGg1a and 34 h for sHt1a. We estimated the surface area of honeybees to be about 34-fold larger than that of varroa mites, and after adding a safety margin applied a 52-fold higher dose of sHt1a and sGg1a to honeybees than the dose applied per varroa mite. When topically applied at 25 μg peptide per honeybee (*n* = 10 worker honeybees per group and 3 repeats per treatment group), neither peptide caused more mortality than the 2% Silwet (v/v) control group (Fig. 3B). This suggests that topical application of sHt1a and sGg1a at equivalent doses for varroa mites and honeybees relative to their respective surface areas, caused *V. destructor* mortality while being safe for honeybees.

### Activity of sHt1a against *Varroadestructor* and honeybees Na_V_ channels

**Fig. 4:**
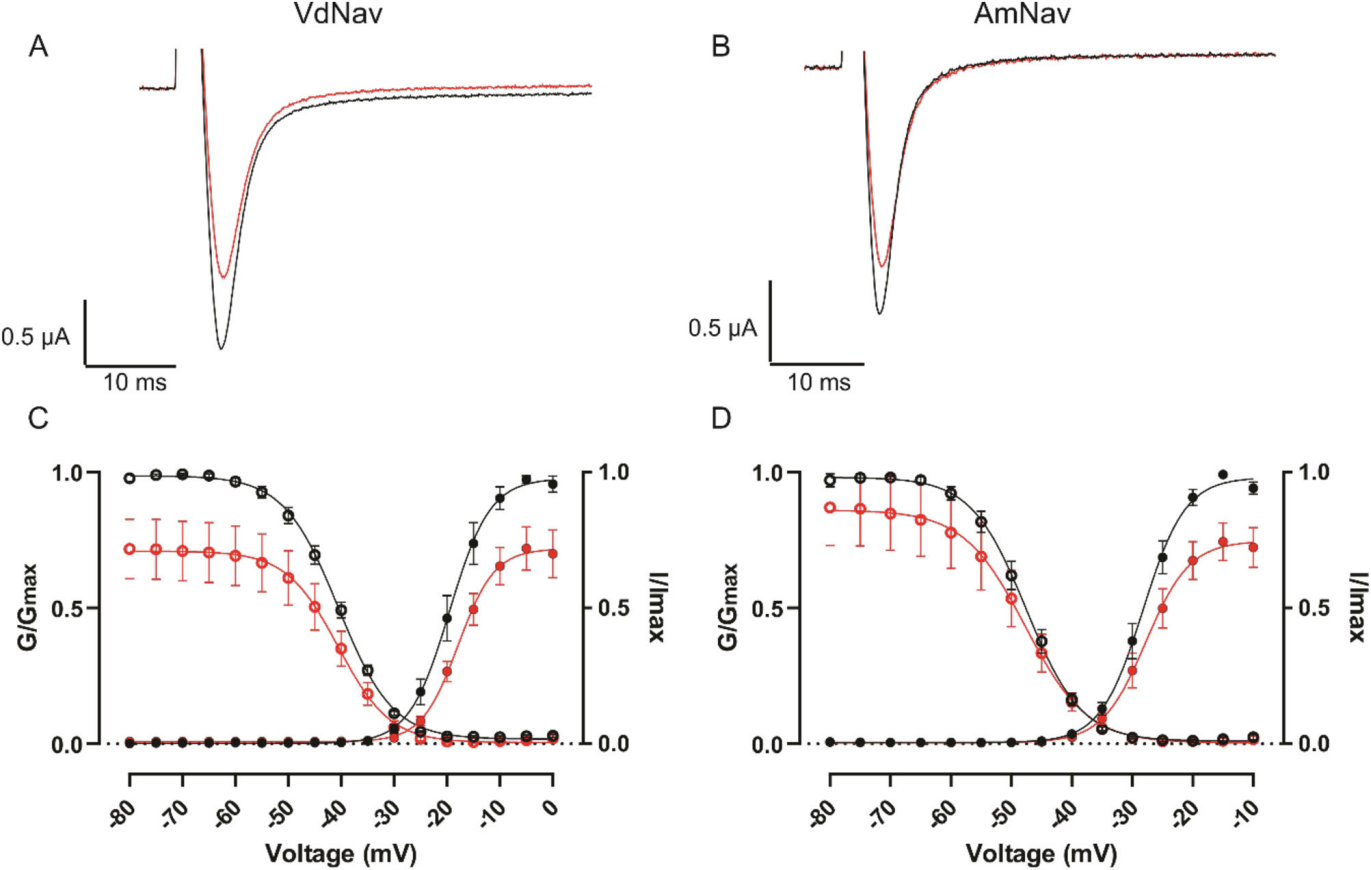
Effect of sHt1a on VdNa_V_and AmNa_V_ channels expressed in *Xenopus laevis* oocytes. **A** Representative current traces for VdNa_V_ before (black) and after (red) sHt1a treatment. **B** Representative current traces for AmNa_V_ before (black) and after (red) sHt1a treatment. **C** Conductance-Voltage (G-V) relationships and channel availability of VdNa_V_ before (black) and after (red) sHt1a incubation. **D** G-V relationships and channel availability of AmNa_V_ before (black) and after (red) sHt1a incubation. All data points are mean ± SEM.

Since many spider-venom peptides target Na_V_ channels^30^, we tested whether this might be the molecular target of sHt1a. Expression of both *V. destructor* (VdNa_V_) and *A. mellifera* (AmNa_V_) sodium channels in *X. laevis* oocytes yielded robust Na^+^ currents within 48–72 h of mRNA injection (Fig. 4A, B, black traces), with half-maximal activation and inactivation values comparable to those previously reported (Fig. 4C, D black traces; Table 2)^31,32^.

**Table 2:** Gating parameters for AmNa_V_ and VdNa_V_ in the presence and absence of sHt1a.

Target	Treatment	Activation		Steady state inactivation		*n* (oocytes)
		V_1/2_ (mV)	Hill slope	V_1/2_ (mV)	Hill slope	
**AmNav**	Control	–28.0 ± 1.0	3.50 ± 0.2	–47.2 ± 1.1	–4.54 ± 0.30	6
	sHt1a	–27.6 ± 1.3	3.62 ± 0.61	–48.2 ± 1.0	–5.12 ± 1.44	6
**VdNav**	Control	–19.0 ± 1.6	3.82 ± 0.37	–40.7 ± 0.8	–5.14 ± 0.22	5
	sHt1a	–17.7 ± 0.7	3.44 ± 0.55	–40.8 ± 0.5	–4.90 ± 1.29	5

All data are mean ± SEM.

Incubation of oocytes with 500 nM sHt1a caused a significant reduction in peak AmNa_V_ (24.7 ± 7.2%, *p* = 0.0095) and VdNa_V_ (25.3 ± 7.8%, *p* = 0.0212) currents (Fig. 4A, B; red traces), without a significant change in the values for half-maximal activation or inactivation values (Fig. 4C, D; red traces; Table 2). The fact that sHt1a was equally active in inhibiting varroa and honeybee Na_V_ channels combined with the lack of effect of sHt1a by topical application in honeybees suggests that either sHt1a more easily penetrates the external barrier of varroa mites than honeybees, or that honeybees have acquired some other form of resistance to Ht1a, such as enzymatic inactivation, thereby providing a safety window for potential varroa treatments. In comparison to other potent insect Na_V_ channel targeting spider toxins, sHt1a is less potent than Tx4(6-1) (IC_50_ = 25 nM^33^) or Dc1a (EC_50_ = 65 nM^34^), but similarly potent to Aps III (IC_50_ = 540 nM^35^) and Jingzhaotoxin-I (IC_50_ = 760 nM^36^). Further testing on other molecular targets is therefore required to determine whether insect Na_V_ channels are the primary target of Ht1a.

### In vitro characterisation of sHt1a and sGg1a against important human receptors and ion channels

We also examined the activity of sHt1a and sGg1a against a range of important molecular targets in vertebrates, including the skeletal muscle Na_V_ channel isoform Na_V_1.4, the cardiac Na_V_ channel isoform Na_V_1.5, the neuronal voltage-gated sodium channels Na_V_1.2/Na_V_1.7, the neuronal voltage-gated calcium channel Ca_V_2.2, and α3-containing nnAChRs. Consistent with the invertebrate-specific activity of both sHt1a and sGg1a, the peptides did not significantly affect Na_V_1.4 and Na_V_1.5 currents (Supplementary Fig. 4) or neuronal Na_V_ or Ca_V_2.2 isoforms (Supplementary Fig. 5A, B). At a very high concentration (10 µM), sHt1a caused partial inhibition of α3 nAChRs (Supplementary Fig. 5C).

In conclusion, a systematic screening of 50 arthropod venoms against varroa mites revealed that acaricidal/varroacidal activity is abundant among arachnid venoms. Fractionation of two of the most potent spider venoms in the present study led to the identification of the peptides Ht1a and Gg1a that were both lethal to varroa mites by topical application but innocuous to honeybees in vivo. The weak or absent activity of Ht1a and Gg1a at key human ion channels and receptors suggests that these peptides are likely invertebrate-specific. Although residue studies in hive products and detailed human off-target and toxicology tests remain to be conducted, our data suggests that topical application of Ht1a and Gg1a by spraying within the beehive could be an effective strategy for targeting *V. destructor* that would be safe for application by beekeepers and well tolerated by the honeybees. The abundance of topically active acaricidal venoms was particularly surprising given that spider venoms are normally delivered via injection^37^. This poses the question about the ecological role of these acaricidal toxins. Given that mites of the order Mesostigmata (which also includes *V. destructor*) are common parasites of spiders^38^, an anti-parasitic role for topically applied venom seems plausible. While the exact mechanism of how these topically applied acaricidal peptides enter the varroa mites remains to be determined, it could provide an interesting case study to further fine-tune some recent definitions of the term “venom”^39,40^.

## Methods

### Venom collection

All arthropod venoms used for this study (Table 1) were isolated by electrical stimulation as previously described^41,42^, then lyophilised and stored at −20 °C prior to use.

### Nomenclature

Peptide toxins were named using the rational nomenclature described previously^29^. Spider taxonomy was taken from the World Spider Catalog Version 27^23^.

### Identification of varroacidal venoms and venom fractions

An outline of the project discovery pipeline is presented in Supplementary Fig. 1. To identify varroacidal venom peptides, we screened venoms for activity against *V. destructor* by topical application. Topical tests were performed in several stages. First, whole venom was tested to determine mortality for *V. destructor*. Hit venoms were then subjected to several fractionation steps to isolate the active compounds. For the initial venom screen, a panel of 50 arthropod venoms (Table 1) were topically applied to *V. destructor* mites. Venoms were dissolved in 90% acetonitrile (v/v), and a 10 μg venom aliquot was applied per mite (*n* = 5 mites per venom). The dorsal plate of *V. destructor* mites (average mass 0.38 mg) was fixed to adhesive tape (ScotchTM 666 3 M; 19 × 32.9 mm, double sided) on microscope slides (ground edges 90° frosted end, 76 × 26 mm). A Nanoject II (Automatic Injector with Glass Capillaries, Drummond Cat.No. 3-000-205 A) was used for topical application of venom and control (90% acetonitrile) solutions. To accelerate solvent evaporation, the total volume of 483 nL was applied in seven aliquots of 69 nL each to the ventral plate of the mites between the posterior pair of legs. Each drop was allowed to dry before applying the next one, with the entire application procedure taking up to 1 h. Mite mortality was determined at various time intervals up to 48 h after the treatment using an Olympus stereomicroscope (SZX10/SZX2 Series). Initially, each mite was examined for movements of the legs, and immobile mites were stimulated with the tip of a wooden toothpick. Detection of static electricity by the mites, a few millimetres before the actual contact with the toothpick, was usually sufficient to trigger movement in mites that were still alive. Mites not responding to this stimulation were touched with the toothpick and considered dead if they did not react to this stimulation. At the later stage of the project, the slides with mites were placed into a self-constructed automated video monitoring system (using a USB camera) that employs an algorithm in OpenCV to subtract images from subsequent frames enabling the detection of changes in pixel intensity due to movements, for example of the extremities. Leg movements were stimulated with an air current applied over the mites at the time of image acquisition to determine mite mortality. The percentage of mite mortality for each venom was calculated at 2, 6 and 24 h, and adjusted by correcting for the mortality of the corresponding control group receiving only 90% acetonitrile.

### Isolation, purification and MS-based sequencing of varroacidal peptides from the active venom fractions

To isolate the active varroacidal venom components, we used reversed-phase high performance liquid chromatography (RP HPLC). Pooled venom from ten female Tasmanian cave spiders (*H. troglodytes*) and venom from a single female Giant Japanese funnel-web spider (*G. gigas*) were fractionated via RP HPLC on a Prominence HPLC system (Shimadzu Scientific Instruments, Rydalmere, NSW, Australia). Venom (1–2 mg dried weight) was loaded onto a C18 RP HPLC column (Phenomenex Jupiter; 250 × 4.6 mm, 5 μm particle size, 250 Å pore size), and fractionated using the following gradients: 5% solvent B (0.1% formic acid (FA) in 90% acetonitrile) in solvent A (0.1% FA in H_2_O) over 5 min, 5–20% solvent B over 5 min, 20–40% solvent B over 40 min, then 40–80% solvent B over 5 min (flow rate 1 mL/min). For *H. troglodytes* venom, the peak containing the active peptide (termed Ht1a) was further purified using a narrow bore C18 column (Vydac; 250 × 2.1 mm, 5 μm) with a flow rate of 0.3 mL/min and using the same solvents as for the initial RP HPLC run. After 5 min at 5% solvent B, a linear gradient of 5–20% solvent B was run over 5 min, followed by 20–-35% solvent B over 45 min and then 35–80% solvent B over 2 min. The varroacidal activity of the purified Ht1a was confirmed using three cohorts of 5 mites, whereas the activity of the purified Gg1a was confirmed using two cohorts of 5 mites.

Mass spectrometry was used to determine the purity of venom fractions and isolated peptide toxins. All molecular masses were determined using matrix-assisted laser desorption/ionisation time-of-flight mass spectrometry (MALDI-TOF MS) using 4700 Triple TOF or 5800 TOF/TOF mass spectrometers (Applied Biosystems, Foster City, CA, USA), or via electrospray ionisation (ESI) MS using a SCIEX 5600 instrument (SCIEX, Framingham, MA, USA). For MALDI-TOF MS, peptide samples were mixed 1:1 (v:v) with α-cyano-4-hydroxy-cinnamic acid matrix (7 mg/mL in 50/50 acetonitrile/H_2_O with 5% FA) and MALDI-TOF spectra were acquired in positive reflector mode. For ESI, peptides were separated on a Zorbax 300SB-C18 column (Agilent Technologies; 2.1 × 150 mm, 3.5 µm) at a flow rate of 180 µL/min using a linear gradient of 1–50% mobile phase B (0.1% FA in 90% acetonitrile) in mobile phase A (0.1% FA) over 9 min on a Shimadzu Nexera UHPLC system coupled to a SCIEX 5600 mass spectrometer. Data were analysed using PeakView software (SCIEX, USA). All reported molecular masses refer to the monoisotopic uncharged peptide unless otherwise stated.

To obtain a partial sequence tag for Ht1a, we used in-source dissociation by MALDI-TOF (MALDI-ISD-MS). 1,5-diaminonaphthalene (1,5-DAN) was used as matrix. Peptide samples were mixed 1:1 (v:v) with 1,5-DAN matrix (20 mg/mL in 50/50 acetonitrile/H_2_O with 5% FA) and MALDI-TOF spectra were acquired in positive reflector mode. Sequencing was performed manually through identification of z- and c-ion series.

Since we did not have access to *G. gigas* specimens for performing venom-gland transcriptomics (see below), we instead employed N-terminal Edman sequencing to determine the sequence of Gg1a (Monash Proteomics & Metabolomics Facility, Melbourne, VIC, Australia). Briefly, about 2.5 µg of Gg1a purified from *G. gigas* venom was applied to a Polybrene pre-treated glass fibre disk and allowed to dry. Cysteine residues were reduced and alkylated by adding a solution containing 0.16% 4-vinylpyridine (Merck) and 0.08% tributylphosphine (Merck) in 80% acetonitrile to the glass fibre disk. The cartridge was then placed without drying into PPSQ-53A Protein Sequencer (Shimadzu), and sequencing via Edman degradation was commenced using the PE Analysis method.

### Venom gland transcriptome of *Hickmania troglodytes* to determine sequence of Ht1a

To obtain the complete sequence of Ht1a, we sequenced a venom-gland transcriptome of the Tasmanian cave spiders (*H. troglodytes*). The venom glands from ten female spiders were extracted via electrostimulation to deplete their glands of venom and thereby stimulate toxin transcription. Three days later, the spiders were anesthetised, then their venom glands were dissected out and placed in TRIzol^®^ reagent (Life Technologies). Total RNA from pooled venom glands was extracted using the standard TRIzol^®^ protocol. mRNA enrichment from total RNA was performed using an Oligotex direct mRNA mini kit (Qiagen). RNA quality and concentration were measured using a Bioanalyzer 2100 pico chip (Agilent Technologies). A cDNA library was constructed from 10 μg mRNA using the standard Roche cDNA rapid library preparation and emulsion PCR. Sequencing was carried at the Australian Genome Research Facility using a ROCHE GS-FLX sequencer. The Raw Standard Flowgram File (.SFF) was converted to Fastq using the sff_extract tool in seq_crumbs (https://github.com/JoseBlanca/seq_crumbs) with default settings. Low-quality sequences were filtered out using a Qual cutoff value of 25. Reads passing QC were visualised using FastQC (www.bioinformatics.babraham.ac.uk/projects/fastqc/). De novo assembly was performed using MIRA software v3.2.1^43^ using the following parameters: -GE:not=4 --project=Htrogodytes --job=denovo,est,accurate,454 454_SETTINGS -CL:qc=no -AS:mrpc=1 -AL:mrs=99,egp=1; this yielded 13,561 contigs. Assembled sequences were manually inspected before submission to the Tox|Note analysis pipeline within ArachnoServer^44^ to identify putative toxin-encoding open reading frames (Tox|Blast and Tox|Seek modules), as well as annotate and submit datasets to ENA (Tox|Name and Tox|Submission modules, respectively). This approach returned 593 putative toxin sequences, which were used as a database for searching the Ht1a sequence tag obtained by MALDI-ISD-MS.

### Chemical synthesis, purification and folding of varroacidal peptides Ht1a and Gg1a

To obtain sufficient amounts of Ht1a and Gg1a to characterise their activity, we used solid-phase peptide synthesis (SPPS). An initial batch of Ht1a was made using Boc SPPS to produce two peptide fragments that were subsequently joined by native chemical ligation (see Supplementary Information for method details). To simplify the synthesis, synthetic Ht1a and Gg1a were subsequently produced by synthesising the full-length peptides using Fmoc SPPS at 0.1 mmol scale using a Liberty Prime microwave-assisted automated peptide synthesiser (CEM, Charlotte, NC, USA). Ht1a/Gg1a were assembled on Fmoc-Leu-Wang resin using the following side-chain protecting groups: Arg (2,2,4,6,7-pentamethyldihydrobenzofuran-5-sulfonyl), Asn (trityl), Asp (O-3-methylpent-3-yl), Glu (tert-butyl ester), Cys (trityl), Lys/Trp (tert-butyloxycarbonyl), and Ser/Thr/Tyr (tert-butyl). Fmoc deprotection was achieved via 25% pyrrolidine/*N*,*N*-dimethylformamide, while Fmoc-amino acids, diisopropylcarbodiimide & Oxyma Pure (1:2:1) were used for couplings (5 molar equivalents) at 105°C. Following synthesis and final Fmoc deprotection, the resin was washed with dichloromethane and subsequently cleaved via incubation with 92.5% trifluoroacetic acid (TFA), 2.5% triisopropylsilane, 2.5% 3,6-dioxa-1,8-octanedithiol, and 2.5% H_2_O at 40 °C for 40 min. Cleaved products were precipitated with ice-cold diethyl ether and subsequently lyophilised with acetonitrile/H_2_O (1:1) prior to preparative RP HPLC purification.

For purification of synthetic Ht1s (sHt1a) and Gg1a (sGg1a), peptides were desalted using a Zorbax 300SB C18 preparative RP HPLC column (Agilent Technologies, 21.2 × 150 mm, 5 μm) and eluted at a flow rate of 24 mL/min using a gradient of 10–60% solvent B over 50 min. Synthetic peptides were further purified using a C4 semi-preparative RP HPLC column (Phenomenex Jupiter, 250 × 10 mm, 10 μm) and a C18 analytical RP HPLC column (Kinetex, 250 × 4.6 mm, 5 μm) (Supplementary Fig. 2). Fractions of similar purity containing the correct peptide mass (as determined via MALDI-TOF MS) were collected, then the peptide solutions were lyophilised.

To obtain the folded, oxidised forms of sHt1a and sGg1a, the purified reduced peptides were dissolved at a final concentration of 0.1 mg/mL in a redox buffer comprised of 0.6 M guanidine hydrochloride (GnHCl), 0.33 M ammonium acetate (NH_4_CH_3_CO_2_), 1.5 mM reduced glutathione, 0.15 mM oxidised glutathione, pH 8 for Ht1a and pH 6.7 for Gg1a. The folding buffer was stirred at 4 °C with exposure to air for 48 h, then the reaction was terminated by addition of neat TFA. Each peptide was desalted via RP HPLC, then complete oxidation was confirmed using MALDI-TOF MS with CHCA as matrix (6 Da decrease in mass compared to the reduced forms).

### Confirmation of varroacidal activity of synthetic Ht1a and Gg1a

The same procedure initially used for identifying varroacidal venoms was used for testing the topical activity of sHt1a and sGg1a. The peptides were dissolved in 0.5% Silwet L-77 (v/v), a silicone-based surfactant that is commonly used as a spreading agent for foliar sprays. 0.48 μg of peptide was topically applied to each mite, with *n* = 5 mites per treatment group. The activity of the sHt1a was confirmed using three cohorts of *n* = 5 mites, whereas the activity of sGg1a was confirmed using four cohorts of *n* = 5 mites. As a negative control, an equal volume of 0.5% Silwet (v/v) was topically applied (four cohorts of *n* = 5 mites), and 3% oxalic acid, a known varroacide used in commercial products^10^ was used as positive control (eight cohorts of *n* = 5 mites). Mite mortality was recorded at 2-hourly intervals between 0 and 48 h after the treatment. For statistical analysis, mite survival data were plotted in Prism 10.0 (GraphPad Software, CA, USA), then a two-way ANOVA followed by Dunnett’s multiple comparisons test was performed, comparing the peptide and oxalic acid treatment against the respective 0.5% Silwet (v/v) control group data.

### Off target testing of synthetic Ht1a and Gg1a against honeybees

If sprayed within a beehive, the varroacidal peptides would need to lethal for *V.* *destructor*, but safe for the honeybees. To evaluate potential off-target activities of the varroacidal peptides, we first needed to quantify how much larger the body surface of the bees is in comparison to the mites to understand how much more of the varroacidal peptides they would receive when being sprayed. Assuming an elliptical body shape of *V. destructor*, the surface area can be calculated according to the following formula:

$${\rm{A}}={\rm{\pi }}\times {\rm{a}}\times {\rm{b}}\times 2$$

with the factor of 2 considering the dorsal and ventral side, where (a) is the semi-major axis (half the length) and (b) is the semi-minor axis (half the width). Using an average adult *V. destructor* size of 1.15 mm in length and 1.7 mm in width, that would then result in:

$${\rm{A}}={\rm{\pi }}\times 0.575{\rm{mm}}\times 0.85{\rm{mm}}\times 2=3.07{{\rm{mm}}}^{2}$$

For honeybees, the exposure surface area has been determined as 1.05 cm^2^/bee (=105 mm^2^)^45^, indicating that bees have a 34-fold larger surface area than the mites. This also means that if we evenly applied these peptides via spraying at a certain concentration to both bees and mites, then the bees would receive a 34-fold higher peptide quantity due to their correspondingly higher surface area.

To allow for an additional safety margin, we topically applied 25 μg of each peptide per bee, which is 52-fold more than the 0.48 μg that each varroa mite received. The effects of sHt1a and sGg1a were tested on honeybees kept in plastic containers in the laboratory. The containers consisted of 250 ml polypropylene round cups with a 52 mm diameter hole cut into the bottom, covered with a 68 mm diameter circle of all-purpose cloth (brand: Peggy Perfect) hot glued to the edge of the hole on the outside of the cup (Supplementary Fig. 3). A 14 mm diameter hole was cut in the lid of the cup to insert a 5 mL syringe which served as feeder. The syringe was filled with 50% sucrose solution, and its tip was cut off to facilitate solution intake by the bees. Honeybee workers were obtained from a comb with emerging individuals placed in an incubator set to 34.5 °C and 70% humidity^46^. The next day, groups of 10 freshly emerged workers were collected, introduced into the prepared cages and placed back in the incubator overnight. The next morning, peptide and control solutions to be tested were freshly prepared from the stock solutions stored at 4 °C. Then, the bees were sedated with CO_2_ gas until they stopped moving. Immediately after all bees stopped moving, the plastic lid was removed, and 15 µL of testing solution (containing 2% Silwet (v/v) or 25 μg of each peptide per bee) was topically applied on each of the 10 bees per group. The solution was applied on the abdomen or thorax, but not on the wings or head of the individuals. Following treatment, the worker bees were allowed to recover from the sedation and the containers with the bees were put back in the incubator, with mortality being manually checked daily. At the end of each experiment, all arthropods were euthanised by freezing at −20 °C.

### Testing for activity of synthetic Ht1a and Gg1a on *Varroa destructor* and honeybee ion channels

To examine the activity of sHt1a against target and off-target arthropod voltage-gated sodium (Na_V_) channels, we used electrophysiological recordings of *V. destructor* (Vd) or *A. mellifera* (Am) Na_V_ channels expressed in *Xenopus laevis* oocytes. The cDNA sequences of the VdNa_V_ (in pGH19; kindly gifted by K. Dong^31^) and AmNa_V_ channel (in pcDNA3.1; Genscript, USA) were confirmed by automated Sanger sequencing (Eurofins Genomics, Germany). RNA was synthesised using T7 polymerase (mMessage mMachine kit, Invitrogen, USA) after linearising the cDNA with an appropriate restriction enzyme (New England Biolabs, USA). Na_V_ channel RNA was microinjected into defolliculated *X. laevis* oocytes (Nasco^®^, USA) which were then incubated for 2–3 days at 17 °C in Barth’s medium (in millimolar (mM): 96 NaCl, 2 KCl, 5 HEPES, 1 MgCl_2_, and 1.8 CaCl_2_ supplemented with 50 µg/mL gentamycin, pH 7.4 adjusted with NaOH (Sigma, USA)). The use of *X. laevis* complied with national and Flemish guidelines adhered to by the Ghent University Animal Care and Use Committee. Electrophysiological characteristics were studied using the two-electrode voltage-clamp technique (OC-725C; Warner Instruments, USA) with a 150-µL recording chamber as previously described^47^. All data were filtered at 4 kHz (tunable active filter model 900, Frequency Devices Inc., USA) and digitised at 20 kHz using pClamp 10 software (Molecular Devices, USA). The external recording solution used was ND-100 (in mM: 100 NaCl, 5 HEPES, 1 MgCl_2_, and 1.8 CaCl_2_, pH 7.6 adjusted with NaOH; chemicals from Sigma, USA). Microelectrode (Narishige^®^ PC-10 vertical puller) resistances were 0.5–1.0 MΩ when backfilled with 3 M KCl. All experiments were performed at room temperature (~22 °C).

For electrophysiological experiments, sHt1a dissolved at 50 µM in ND100 was added to the recording chamber containing ND100 to a final concentration of 500 nM. After current stabilisation, normalized conductance-voltage (G-V) and channel availability relationships were obtained by measuring steady-state currents upon stepwise depolarisation for 50 ms from a holding potential of –90 mV in 5 mV increments, followed by a 50 ms pulse at –20 mV. The data were fitted with a single Boltzmann function according to *I*/*I*_max_ (or G/G_max_) = [1+exp(-zF(V-V_1/2_)/RT)]^−1^, where *I*/*I*_max_ is the normalised current amplitude, z is the equivalent charge, *V*_1/2_ is the half-activation voltage, *F* is Faraday’s constant, *R* is the gas constant, and *T* is the temperature in Kelvin. Off-line data analysis was performed using Clampfit 10 (Molecular Devices, USA), Excel (Microsoft Office) and Prism 10 (GraphPad). Tests for statistical differences included a student’s *t*-test for *V*_1/2_ values and a Kruskal–Wallis test for current inhibition, with *p* < 0.05 considered to be significant.

### Electrophysiological testing of sHt1a and sGg1a on human receptors

To examine for off-target activity on mammalian ion channels and receptors, we first tested the activity of sHt1a and Gg1a against human Na_V_ channels. Whole-cell patch-clamp recordings were performed on Human Embryonic Kidney (HEK) 293 cells stably expressing human Na_V_1.4 or Na_V_1.5 in conjunction with the β1 subunit (SB Drug Discovery, Glasgow, United Kingdom) using a QPatch II automated electrophysiology platform (Sophion Bioscience, Ballerup, Denmark). Whole-cell currents were filtered at 8 kHz and acquired at 25 kHz. The linear leak was corrected by P/4 subtraction (leak potential −90 mV, leak sweep amplitude 10%). Series resistance across recorded cells ranged between 5 and 10 MΩ. The extracellular solution (ECS) consisted of (in mM): 145 NaCl, 4 KCl, 2 CaCl_2_, 1 MgCl_2_, 10 HEPES, and 10 glucose, adjusted to pH 7.4 with NaOH and to 305 mOsm/L with sucrose. The intracellular solution (ICS) consisted of (in mM): 140 CsF, 1 EGTA, 5 CsOH, 10 HEPES, and 10 NaCl, adjusted to pH 7.3 with CsOH and 320 mOsm/L with sucrose. Currents were elicited from a holding potential of −90 mV delivered with a 50 ms depolarising pulse to −20 mV every 20 s (0.05 Hz). Peptides were diluted in ECS containing 0.1% BSA and applied to cells for at least 2 min at the concentrations stated. Peak currents were normalised to the buffer control.

To further test the selectivity of sHt1a and sGg1a, we examined their activity on hNa_V_1.2/Na_V_1.7 and hCa_V_2.2 channels, as well as the hα3β2/4 nicotinic acetylcholine receptor (nAchR), that are endogenously expressed in the neuroblastoma cell line SH-SY5Y using a high-throughput fluorescent assay as previously described^48^. In brief, SH-SY5Y cells were seeded at a density of 40,000 cells/well in black-walled imaging plates (Corning, Lowell, MA, USA), then incubated for 48 h at 37 °C in a 5% humidified CO_2_ incubator. On the day of the assay, cells were loaded with Calcium 4 No-wash dye (Molecular Devices) by reconstituting the lyophilised dye in physiological salt solution (PSS in mM: 140 NaCl, 11.5 glucose, 5.9 KCl, 1.4 MgCl_2_, 1.2 NaH_2_PO_4_, 5 NaHCO_3_, 1.8 CaCl_2_, 10 HEPES, pH 7.4). Cells were incubated with the dye for 30 min at 37 °C in a 5% humidified CO_2_ incubator. Gg1a and Ht1a (10 µM) were added 5 min prior to stimulation with either veratridine (50 µM, hNa_V_1.2/Na_V_1.7 activator), nicotine (30 µM, hα3β2/4 nAchR activator) or KCl/CaCl_2_ (90 mM/5 mM, Ca_V_1.3/2.2 activator) Fluorescent responses were recorded using a FLIPR^Penta^ fluorescent plate reader (Molecular Devices) with excitation at 470–495 nm and emission at 515–575 nm.

## Supplementary information

Supplementary Information

## Data Availability

All data generated or analysed during this study are included in this published article and its supplementary information files. Metadata and annotated nucleotide sequences were deposited in the European Nucleotide Archive under project accessions: PRJEB15860.

## References

[1] Gallai, N., Salles, J. M., Settele, J. & Vaissière, B. E. Economic valuation of the vulnerability of world agriculture confronted with pollinator decline. *Ecol. Econ.* **68**, 810–821 (2009).

[2] Klein, A. M. et al. Importance of pollinators in changing landscapes for world crops. *Proc. Biol. Sci.* **274**, 303–313 (2007). 17164193 10.1098/rspb.2006.3721 PMC1702377

[3] Aizen, M. A. & Harder, L. D. The global stock of domesticated honey bees is growing slower than agricultural demand for pollination. *Curr. Biol.* **19**, 915–918 (2009). 19427214 10.1016/j.cub.2009.03.071

[4] Garibaldi, L. A. et al. Exploring connections between pollinator health and human health. *Philos. Trans. R. Soc. Lond. B Biol. Sci.* **377**, 20210158 (2022). 35491592 10.1098/rstb.2021.0158 PMC9058530

[5] Mashilingi, S. K., Zhang, H., Garibaldi, L. A. & An, J. Honeybees are far too insufficient to supply optimum pollination services in agricultural systems worldwide. *Agric. Ecosyst. Environ.* **335**, 108003 (2022).

[6] Goulson, D., Nicholls, E., Botias, C. & Rotheray, E. L. Bee declines driven by combined stress from parasites, pesticides, and lack of flowers. *Science* **347**, 1255957 (2015). 25721506 10.1126/science.1255957

[7] Rosenkranz, P., Aumeier, P. & Ziegelmann, B. Biology and control of *Varroa destructor*. *J. Invert. Pathol.* **103**, S96–S119 (2010). 10.1016/j.jip.2009.07.016 19909970

[8] Wilfert, L. et al. Deformed wing virus is a recent global epidemic in honeybees driven by Varroa mites. *Science* **351**, 594–597 (2016). 26912700 10.1126/science.aac9976

[9] Chapman, N. C. et al. The final frontier: ecological and evolutionary dynamics of a global parasite invasion. *Biol. Lett.* **19**, 20220589 (2023). 37222245 10.1098/rsbl.2022.0589 PMC10207324

[10] Jack, C. J. & Ellis, J. D. Integrated pest management control of *Varroa destructor* (Acari: Varroidae), the most damaging pest of (*Apis mellifera* L. (Hymenoptera: Apidae)) colonies. *J. Insect Sci.* **21**, 6 (2021). 34536080 10.1093/jisesa/ieab058 PMC8449538

[11] Bahreini, R., Docherty, C., Feindel, D. & Muirhead, S. Comparing the efficacy of synthetic Varroacides and Varroa destructor phenotypic resistance using Apiarium and Mason jar bioassay techniques. *Pest Manag. Sci.* **80**, 1577–1592 (2024). 37974358 10.1002/ps.7891

[12] Maggi, M. D., Ruffinengo, S. R., Damiani, N., Sardella, N. H. & Eguaras, M. J. First detection of *Varroa destructor* resistance to coumaphos in Argentina. *Exp. Appl. Acarol.* **47**, 317–320 (2009). 19009360 10.1007/s10493-008-9216-0

[13] Rodrıguez-Dehaibes, S., Otero-Colina, G., Sedas, V. & Jimenez, J. Resistance to amitraz and flumethrin in *Varroa destructor* populations from Veracruz, Mexico. *J. Apic. Res.* **44**, 124–125 (2015).

[14] Dawdani, S. et al. Effects of dialkoxybenzenes against *Varroa destructor* and identification of 1-allyloxy-4-propoxybenzene as a promising acaricide candidate. *Sci. Rep.* **13**, 11195 (2023). 37433810 10.1038/s41598-023-38187-6 PMC10336134

[15] Dietemann, V. et al. *Varroa destructor*: research avenues towards sustainable control. *J. Apicult. Res*. **51** (2012).

[16] Lu, R. X., Ibrahim, A., Rueppell, O., Plettner, E. & Pernal, S. F. Field trials of the novel varroacide, 1-allyloxy-4-propoxybenzene, against *Varroa destructor* in Western Canada. *Sci. Rep.* **15**, 40183 (2025). 41249249 10.1038/s41598-025-23935-7 PMC12623769

[17] Herzig, V. Arthropod assassins: crawling biochemists with diverse toxin pharmacopeias. *Toxicon* **158**, 33–37 (2019). 30496730 10.1016/j.toxicon.2018.11.312

[18] Michalek, O. et al. Composition and toxicity of venom produced by araneophagous white-tailed spiders (Lamponidae: *Lampona sp*.). *Sci. Rep.* **12**, 21597 (2022). 36517485 10.1038/s41598-022-24694-5 PMC9751281

[19] Polis, G. A. & McCormick, S. J. Scorpions, spiders and solpugids: predation and competition among distantly related taxa. *Oecologia* **71**, 111–116 (1986). 28312091 10.1007/BF00377328

[20] Mukherjee, A. K., Sollod, B. L., Wikel, S. K. & King, G. F. Orally active acaricidal peptide toxins from spider venom. *Toxicon* **47**, 182–187 (2006). 16330063 10.1016/j.toxicon.2005.10.011

[21] Fletcher, J. I. et al. The structure of a novel insecticidal neurotoxin, ω-atracotoxin-HV1, from the venom of an Australian funnel web spider. *Nat. Struct. Biol.* **4**, 559–566 (1997). 9228949 10.1038/nsb0797-559

[22] Nakasu, E. Y. et al. Novel biopesticide based on a spider venom peptide shows no adverse effects on honeybees. *Proc. Biol. Sci.* **281**, 20140619 (2014). 24898372 10.1098/rspb.2014.0619 PMC4071547

[23] World Spider Catalog. *World Spider Catalog* http://wsc.nmbe.ch (2026).

[24] Rein, J. O. *The Scorpion Files* https://www.ntnu.no/ub/scorpion-files/ (2026).

[25] Pineda, S. S. et al. Structural venomics reveals evolution of a complex venom by duplication and diversification of an ancient peptide-encoding gene. *Proc. Natl. Acad. Sci. USA* **117**, 11399–11408 (2020). 32398368 10.1073/pnas.1914536117 PMC7260951

[26] King, G. F. & Hardy, M. C. Spider-venom peptides: structure, pharmacology, and potential for control of insect pests. *Annu. Rev. Entomol.* **58**, 475–496 (2013). 23020618 10.1146/annurev-ento-120811-153650

[27] Saez, N. J. & Herzig, V. Versatile spider venom peptides and their medical and agricultural applications. *Toxicon* **158**, 109–126 (2019). 30543821 10.1016/j.toxicon.2018.11.298

[28] King, G. F. Tying pest insects in knots: the deployment of spider-venom-derived knottins as bioinsecticides. *Pest Manag. Sci.* **75**, 2437–2445 (2019). 31025461 10.1002/ps.5452

[29] King, G. F., Gentz, M. C., Escoubas, P. & Nicholson, G. M. A rational nomenclature for naming peptide toxins from spiders and other venomous animals. *Toxicon* **52**, 264–276 (2008). 18619481 10.1016/j.toxicon.2008.05.020

[30] Klint, J. K. et al. Spider-venom peptides that target voltage-gated sodium channels: pharmacological tools and potential therapeutic leads. *Toxicon* **60**, 478–491 (2012). 22543187 10.1016/j.toxicon.2012.04.337

[31] Du, Y., Nomura, Y., Liu, Z., Huang, Z. Y. & Dong, K. Functional expression of an arachnid sodium channel reveals residues responsible for tetrodotoxin resistance in invertebrate sodium channels. *J. Biol. Chem.* **284**, 33869–33875 (2009). 19828457 10.1074/jbc.M109.045690 PMC2797157

[32] Gosselin-Badaroudine, P. et al. Characterization of the honeybee AmNa_V_1 channel and tools to assess the toxicity of insecticides. *Sci. Rep.* **5**, 12475 (2015). 26202396 10.1038/srep12475 PMC4894402

[33] de Lima, M. E. et al. The toxin Tx4(6-1) from the spider Phoneutria nigriventer slows down Na(+) current inactivation in insect CNS via binding to receptor site 3. *J. Insect Physiol.* **48**, 53–61 (2002). 12770132 10.1016/s0022-1910(01)00143-3

[34] Bende, N. S. et al. A distinct sodium channel voltage-sensor locus determines insect selectivity of the spider toxin Dc1a. *Nat. Commun.* **5**, 4350 (2014). 25014760 10.1038/ncomms5350 PMC4115291

[35] Bende, N. S. et al. The insecticidal neurotoxin Aps III is an atypical knottin peptide that potently blocks insect voltage-gated sodium channels. *Biochem. Pharmacol.* **85**, 1542–1554 (2013). 23473802 10.1016/j.bcp.2013.02.030 PMC3654253

[36] Xiao, Y. C. et al. Jingzhaotoxin-I, a novel spider neurotoxin preferentially inhibiting cardiac sodium channel inactivation. *J. Biol. Chem.* **280**, 12069–12076 (2005). 15548530 10.1074/jbc.M411651200

[37] Lüddecke, T., Herzig, V., von Reumont, B. M. & Vilcinskas, A. The biology and evolution of spider venoms. *Biol. Rev. Camb. Philos. Soc.* **97**, 163–178 (2022). 34453398 10.1111/brv.12793

[38] Masan, P., Simpson, C., Perotti, M. A. & Braig, H. R. Mites parasitic on Australasian and African spiders found in the pet trade; a redescription of *Ljunghia pulleinei* Womersley. *PLoS ONE* **7**, e39019 (2012). 22720019 10.1371/journal.pone.0039019 PMC3374770

[39] Herzig, V. ‘Venom’ - a manipulative weapon for overcoming the victim’s protective barriers. *Trends Ecol. Evolut.* **40**, 1044–1045 (2025). 10.1016/j.tree.2025.09.011 40983564

[40] Jenner, R. A., Casewell, N. R. & Undheim, E. A. B. What is animal venom? Rethinking a manipulative weapon. *Trends Ecol. Evolut.* **40**, 852–861 (2025). 10.1016/j.tree.2025.05.009 40544034

[41] Guo, S., Herzig, V. & King, G. F. Dipteran toxicity assays for determining the oral insecticidal activity of venoms and toxins. *Toxicon* **150**, 297–303 (2018). 29920256 10.1016/j.toxicon.2018.06.077

[42] Walker, A. A. et al. The assassin bug *Pristhesancus plagipennis* produces two distinct venoms in separate gland lumens. *Nat. Commun.* **9**, 755 (2018). 29472578 10.1038/s41467-018-03091-5 PMC5823883

[43] Chevreux, B. et al. Using the miraEST assembler for reliable and automated mRNA transcript assembly and SNP detection in sequenced ESTs. *Genome Res.* **14**, 1147–1159 (2004). 15140833 10.1101/gr.1917404 PMC419793

[44] Pineda, S. S. et al. ArachnoServer 3.0: an online resource for automated discovery, analysis and annotation of spider toxins. *Bioinformatics* **34**, 1074–1076 (2017). 10.1093/bioinformatics/btx661 29069336

[45] Poquet, Y. et al. A pragmatic approach to assess the exposure of the honey bee (*Apis mellifera*) when subjected to pesticide spray. *PLoS ONE* **9**, e113728 (2014). 25412103 10.1371/journal.pone.0113728 PMC4239102

[46] Williams, G. R. et al. Standard methods for maintaining adult *Apis mellifera* in cages under in vitro laboratory conditions. *J. Apic. Res.* **52**, 1–36 (2013).

[47] de Cassia Collaco, R. et al. Anxiety and dysautonomia symptoms in patients with a Na_V_1.7 mutation and the potential benefits of low-dose short-acting guanfacine. *Clin. Auton. Res.* **34**, 191–201 (2024). 38064009 10.1007/s10286-023-01004-1 PMC11805752

[48] Maxwell, M. J. et al. A bivalent remipede toxin promotes calcium release via ryanodine receptor activation. *Nat. Commun.* **14**, 1036 (2023). 36823422 10.1038/s41467-023-36579-w PMC9950431

