# Supplementary material for: Spider venom peptides Ht1a and Gg1a are toxic to honeybee parasite Varroa destructor by topical application
Source: NPJ Drug Discov. 2026 Jun 3;3:16. doi: 10.1038/s44386-026-00050-9 (PMC13233288; doi:10.1038/s44386-026-00050-9)
Supplement: Supplementary file 1 — Supplementary Information [file 44386_2026_50_MOESM1_ESM.pdf]

Supplementary Information

Title

Spider venom peptides Ht1a and Gg1a are toxic to honeybee parasite *Varroa destructor* by topical application

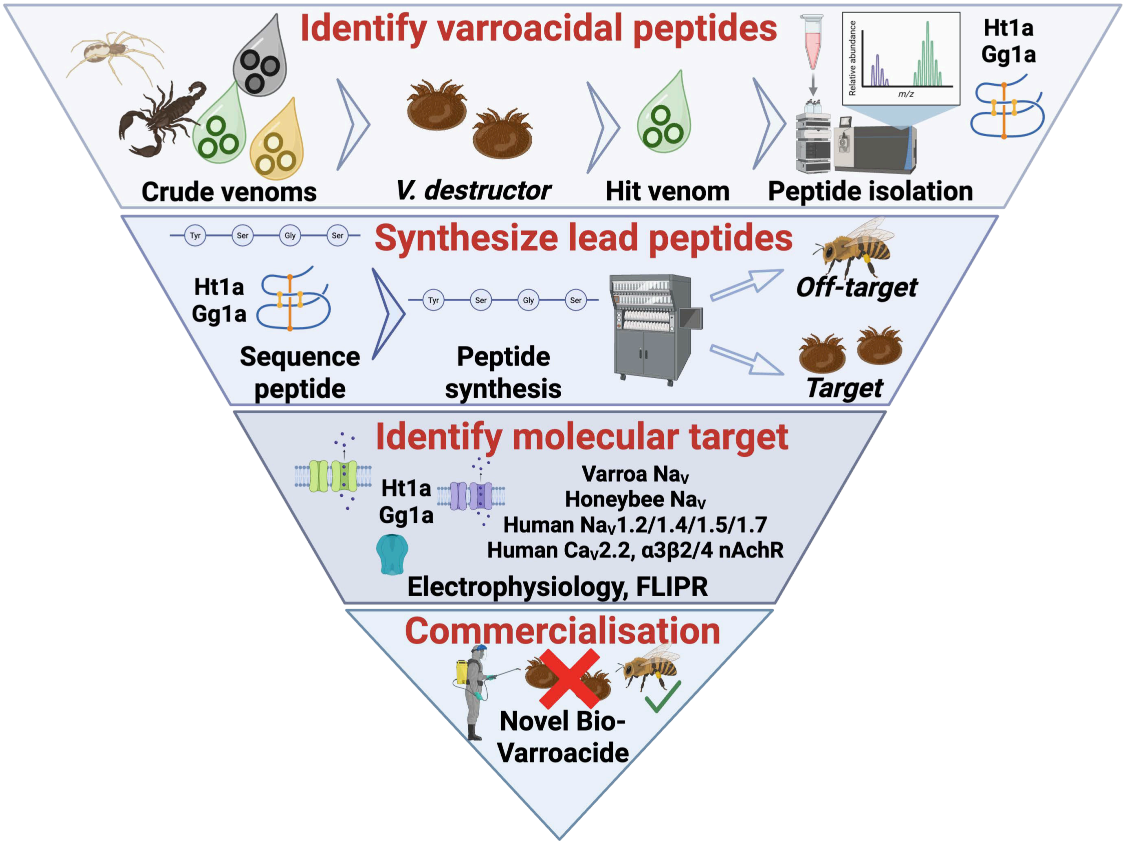

Supplementary Figure 1: Schematic illustration of varroacidal peptide discovery pipeline. Initially, 50 arthropod venoms were screened for topical activity in *Varroa destructor* mites, resulting in the identification of the two varroacidal peptides Ht1a and Gg1a. Synthetic Ht1a and Gg1a were produced by solid phase peptide synthesis and induced topical mortality in *V. destructor* mites but not in *Apis mellifera* honeybees. Electrophysiology and FLIPR were employed to characterise molecular targets of Ht1a and Gg1a, which at 500 nM inhibited ~25% of peak current of varroa and honeybee Na_V channels, while Ht1a at a 10 µM caused partial inhibition of human α3 nAChRs. Future steps towards commercializing these varroacidal peptide candidates include in-hive spraying of varroa-infested honeybee colonies, as well as more extensive off-target testing, establishing economical large-scale production capabilities and optimizing delivery strategies. Figure created with BioRender.com.

Chemical synthesis of Ht1a using native chemical ligation

An initial batch of Ht1a was produced by SPPS of two peptide fragments that were joined by native chemical ligation according to the following method:

Chemical abbreviations

DIEA, *N*,*N*-diisopropylethylamine; DMF, *N*,*N*-dimethylformamide; HBTU, 2-(1H-benzotriazol-1-yl)-1,1,3,3-tetramethyluronium hexafluorophosphate; MPAA, 4-mercaptophenyacetic acid; TCEP, tris(2-carboxyethyl)phosphine; TFA, trifluoroacetic acid; TIPS, triisopropylsilane

Peptide synthesis

A C-terminal fragment (residues 23–49; CAYPNYGLGYYLDSEFFGKRKICSWRL) and N-terminal thioester fragment (residues 1–22; KYCAPRKAVCNTPADCCDKSSR-COSR) of Ht1a were assembled using manual Boc protocols on a 0.25 mmol scale. Boc deprotections were achieved using neat trifuoroacetic acid (TFA) (2 x 1 min). Couplings were performed in DMF using 4 equivalents of Boc-amino acid/HBTU/DIEA (8:8:11) relative to resin loading for a minimum of 10 min. Amino acid side-chains were protected as Asn(Xan), Asp(OcHxl), Arg(Tos), Cys(Meb), Glu(OcHxl), Lys(2-ClZ), Ser(Bzl), Thr(Bzl), Trp(CHO) and Tyr(2-BrZ). For the synthesis of the N-terminal thioester fragment, S-trityl-3-mercaptopropionic acid was first coupled to Leu-OCH2-PAM polystyrene resin then the S-trityl group removed by treatment with 5% TIPS/TFA for 2 × 1 min. The first amino acid of the sequence (Arg) was coupled to the resultant free thiol to form the thioester. Upon completion of assembly of the 23-49 fragment, the formyl side-chain protecting group of Trp was removed using 20% piperidine/5% H_2O/DMF (2 × 30 min). Cleavage from the resin and removal of side-chain protecting groups was achieved by treatment with HF/p-cresol (9:1 v/v) at 0°C for 1 h. After evaporation of HF, the products were precipitated and washed with cold ethanol and lyophilised from 50% acetonitrile/0.1% TF /H_2O. The crude products were purified using preparative RP HPLC. ESI-MS (m/z): Ht1a 23–49 calc. (avg) 1084.6 [M+3H]3+, found 1084.5; Ht1a 1–22-thioester calc. (avg) 873.7 [M+3H]3+, found 873.5.

Native chemical ligation

Purified 1–22-thioester (87 mg) and 23–49 (86 mg) fragments were combined and stirred in a deoxygenated solution of 6 M GnHCl/0.2 M sodium phosphate/20 mM TCEP/50 mM MPAA (pH 7.0, 13 mL) for 16 h at room temperature under an argon atmosphere. The single major product, hexathiol Ht1a 1–49, was isolated using preparative RP HPLC (61 mg). ESI-MS (m/z): calc. (avg) 1413.4 $[M+4H]^{4+}$, found 1413.4.

Oxidative folding of sHt1a

Purified reduced peptide (56 mg), reduced glutathione (100 equiv) and oxidised glutathione (10 equiv) were dissolved in 6 M GnHCl (50 mL) then added to a solution of 0.36 M NH4OAc (pH 8.0, 550 mL) and stirred at 4°C with exposure to air for 48 h. The single major product was isolated using preparative RP HPLC (26 mg). ESI-MS (m/z): calc. 1411.9 (avg) $[M+4H]^{4+}$, found 1411.8.

Reagents and equipment

Solvents for RP HPLC consisted of 0.05% TFA/H_2O (A) and 90% acetonitrile/0.043% TFA/H_2O (B). Analytical HPLC was performed on a Shimadzu LC20AT system using a Thermo Hypersil GOLD C18 column (2.1 x 100 mm) heated at 40°C. Peptide was eluted at a flow rate of 0.3 mL / min using a gradient of 10 to 55% B over 30 min, with detection at 214 nm. Preparative RP HPLC was performed using a Vydac 218TP1022 column running at a flow rate of 16 mL / min using a gradient of 10 to 50% B over 40 min. Mass spectrometry was performed on an API 150 (ABI Sciex) mass spectrometer in positive ion mode. All reagents were obtained commercially and were used without further purification.

Chemical synthesis of sHt1a and sGg1a using Fmoc SPPS chemistry

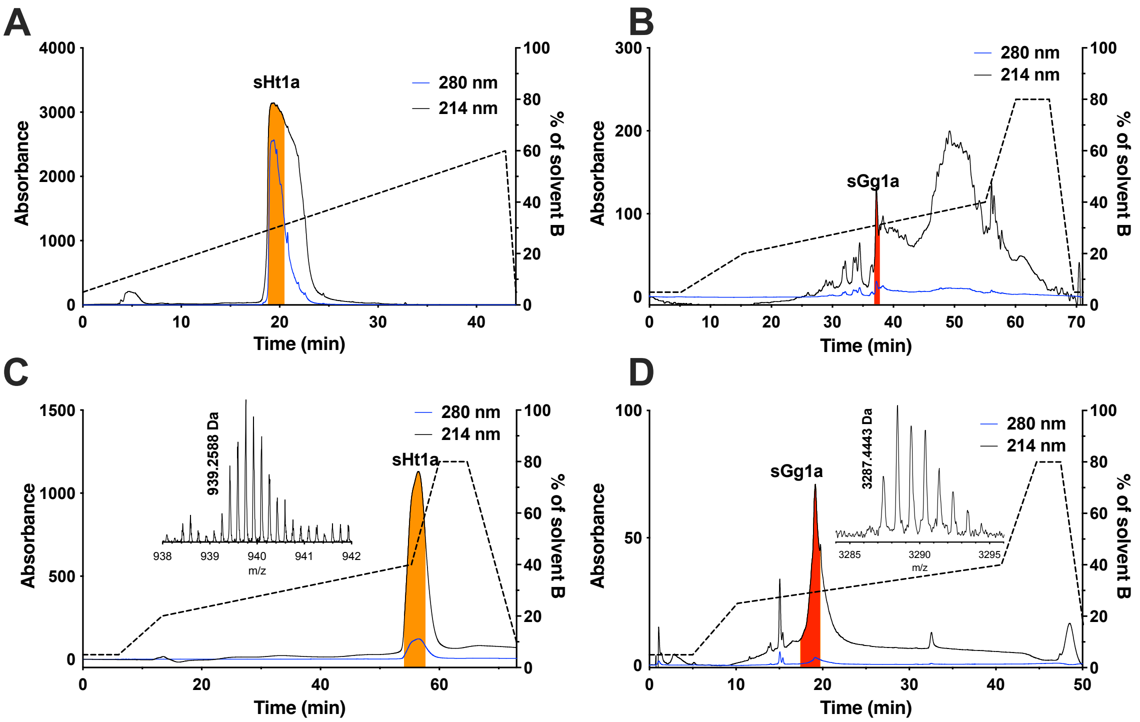

Supplementary Figure 2: RP HPLC chromatograms showing purification of synthetic Ht1a and Gg1a. (**A,B**) Initial purification of Ht1a (**A**) using a C4 RP-HPLC column (250 × 10 mm, 10 µm), and Gg1a (**B**) using a C18 RP HPLC column (Kinetex, 250 × 4.6 mm, 5 µm). (**C,D**) Final purification of Ht1a (**C**) and sGg1a (**D**) using a C_{18} RP HPLC column (Kinetex, 250 × 4.6 mm, 5 µm), but with different gradients. Insets are ESI mass spectrum showing the Ht1a $[M + 6H]^{6+}$ ion (**C**), and MALDI-MS spectrum showing the Gg1a $[M+ H]^{+}$ion. The solid blue and black lines represent the absorbance at 280 nm and 214 nm, while the dashed lines indicate the gradient of solvent B. Fractions that contain Ht1a or Gg1a are highlighted with orange or red, respectively.

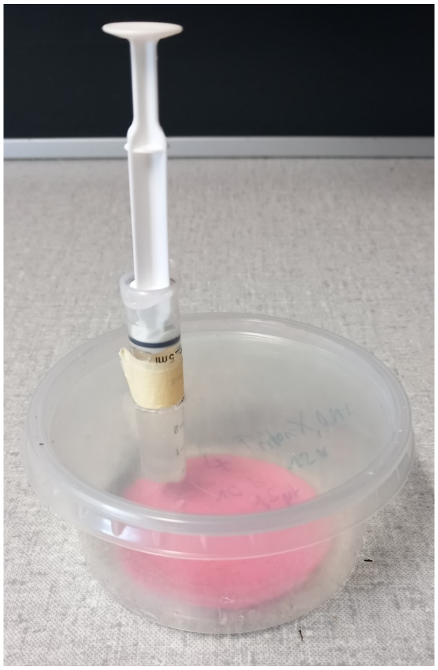

Supplementary Figure 3: Polypropylene container used for testing spider-venom peptides against honeybees.

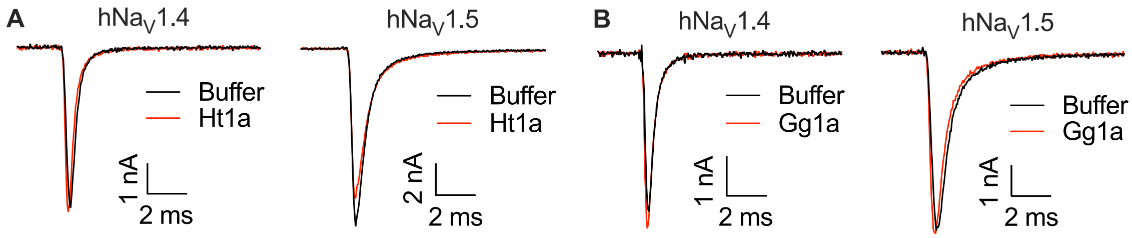

Supplementary Figure 4: **(A)** Representative hNav1.4 and hNav1.5 current traces before (black) and after (red) addition of 3 µM sHt1a. Currents were elicited by a 50 ms pulse to −20 mV from a holding potential of −90 mV. **(B)** Representative hNav1.4 and hNav1.5 current traces before (black) and after (red) addition of 3 µM sGg1a. Currents were elicited by a 50 ms pulse to −20 mV from a holding potential of −90 mV.

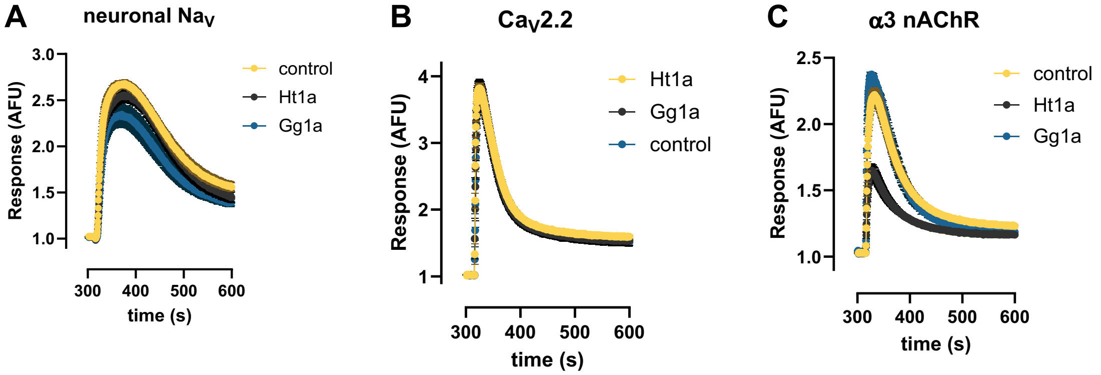

Supplementary Figure 5: Ca^{2+} responses of human neuronal SH-SY5Y cells in the presence of sHt1a (10 µM) and sGg1a (10 µM) in response to stimulation with **(A)** the neuronal Nav channel activator veratridine, **(B)** the Cav2.2 activator KCl, and **(C)** the α3 nAChR activator nicotine.
